# Environmental Goals Addressed in Assessments of Contaminated Sediments

**DOI:** 10.1002/ieam.4223

**Published:** 2019-12-19

**Authors:** Peter Bruce, Yvonne Ohlsson

**Affiliations:** ^1^ Stockholm University Stockholm Sweden; ^2^ Swedgeo Stockholm Sweden

**Keywords:** Sustainable development goals, Environmental risk assessment, Contaminated sediments, Guidelines, Environmental policy

## Abstract

Environmental management often aims at sustainability and at implementing goals such as the United Nations Sustainable Development Goals (SDGs). Several of these goals rely upon healthy sediments, implying that contaminated sediments need to be addressed in environmental management. In Sweden, the SDGs and national environmental objectives are meant to be central in environmental management. However, in environmental risk assessment (ERA) for contaminated sediments, it is not clear to assessors or authorities in Sweden which goals to consider and how. In the present study, we explore which environmental goals ERAs of contaminated sediment should consider, according to representatives from Swedish regulatory and advisory authorities, and to what extent ERAs consider those goals in practice in Sweden. The identified goals indicated that protection of aquatic ecosystems and associated services is a main priority for the authorities. The ERAs analyzed showed that most identified goals were addressed, albeit not consistently. Furthermore, risk assessors seem to put less weight on ecological aspects than authorities do. This indicates that authorities need to stress the importance of environmental aspects and provide guidance on how to address them. Several environmental goals were found to be directly relevant to contaminated sediments and ERAs can contribute to their fulfillment, given that ERA is a basis for management decisions. However, practitioners need clearer guidance on which goals to address and how. Finally, there seems to be good potential for implementation of the SDGs in environmental management in Sweden, due to existing legislation based on sustainable development and well‐anchored national environmental objectives that overlap with the SDGs. *Integr Environ Assess Manag* 2019;00:1–12. © 2019 The Authors. *Integrated Environmental Assessment and Management* published by Wiley Periodicals, Inc. on behalf of Society of Environmental Toxicology & Chemistry (SETAC)

## KEY POINTS

- Sweden aims at taking the lead toward the sustainable development goals (SDGs), yet for environmental risk assessments (ERAs) of contaminated sediments the authorities do not yet require assessors to address such goals; neither are there any official guidelines on how to address them.
- We have identified a set of environmental objectives and SDGs that authorities consider directly relevant for stakeholders to address when assessing risks from contaminated sediments.
- The set of goals indicates that the scope of ERA in Sweden has to be broader to include sustainable management of ecological functions and ecosystem services in addition to the prevalence of contaminants and human health risks.
- This study indicates that ERA in a Swedish context is in a good position to potentially contribute more to several of the goals identified as relevant than is contributed today, with guidance and cooperation with the authorities.

## INTRODUCTION

As a society, we have many goals that commit us to a more sustainable development. A global example is the set of sustainable development goals (SDGs) ratified by all member states in the United Nations. The SDGs represent the member states' intention to make the world resilient and sustainable in areas that are considered to be of critical importance for both society and the environment at large (UN 2015). However, it is not always clear how and when environmental managers should implement the goals in practice.

The Swedish government describes the SDGs as the agenda for planning environmental management (SEEPA et al. 2018; Swedish Government Offices 2018). It is actually Sweden's explicit intention to take the lead in sustainable development and management of the environment (Swedish Government Offices 2018). Therefore, it could be expected that the SDGs would be addressed in Swedish environmental management.

Sweden's marine and freshwater bodies cover 120 000 km^2^ (Statistics Sweden 2011), but marine and freshwater sediments are still often overlooked in environmental management. Sediment processes and functions provide important ecosystem services, such as filtering and storing excess nutrients, supplying food, and supporting recreational values (Troell et al. 2005; Schmidt et al. 2011). Given the functions and services they provide, aquatic ecosystems should be carefully managed when they are at risk in order to fulfill several of the environmental and development goals. Sediments along the Swedish coast have higher levels of contamination than other coastal areas in the region (Borg and Jonsson 1996; Berggren et al. 1999; Jonsson 2000). Sediments in many lakes and streams in Sweden are contaminated from historical activities. The present study investigates which environmental and development goals ought to be addressed in management of contaminated sediments and to what extent they are already considered in practical applications today, specifically in environmental risk assessments (ERAs) that are important as the basis for management decisions.

## BACKGROUND

Several countries in Europe, as well as the USA and Canada, use ERA to assess and help manage risks associated with contaminated sediments (den Besten et al. 2003; Anderson et al. 2008; Suter 2008). The risks from contaminated sediments are often complex to estimate, and methodology and strategies often differ from those suitable for contaminated land. Frameworks and guidelines for ERA have therefore been developed specifically for sediments (e.g., Chapman et al. 2002). In general, ERA follows the following phases: problem formulation, analysis, and risk characterization. The problem formulation defines which objectives the ERA strives toward, which endpoints to protect, and which endpoints to assess, and it identifies possible stressors and sources. The analysis phase provides the information needed to determine the environmental response to the stressor and exposure of interest. During risk characterization, the assessors interpret the results from the analysis to estimate the risk to the assessment endpoints and describe the probability and level of adverse effects to determine the need for risk mitigation.

Environmental risk assessment provides an effective basis for decision making (Barnthouse 2008). However, in Sweden, there is no official guidance for how to perform ERA specifically for contaminated sediments (Severin et al. 2018). The focus within contaminated site management has until now been more on contaminated terrestrial areas than on aquatic environments. Management of contaminated sites in Sweden is closely related to a national inventory that systematically identified and registered mainly land‐based industries that may have caused contamination (SEEPA 1999, 2014). When site investigations were initiated nationally, they tended to focus on wood preservation sites and other sites where soil contamination was important. Subsequently, investigations focused on dry‐cleaning sites and sites with groundwater contamination. Very few of the early site investigations focused on contaminated sediments. Now, Swedish stakeholders experience a need for standardized guidance for sediment ERA (Ländell et al. 2014).

In the present study, we have investigated to what extent ERAs of contaminated sediments currently implement relevant SDGs and other environmental and development goals. Conducting an ERA often takes several years, and because the SDGs were recently ratified in 2015 it is too early to see their implementation directly in ERA. However, in Sweden the ecological dimension of the SDGs is supposed to be implemented through the Swedish National Environmental Objectives (NEOs) (SEEPA et al. 2018). The NEOs are a set of national environmental goals that have been in place long enough (since 1999) for us to expect them to have been considered in ERAs conducted in recent years. The NEOs have a large overlap with the environmental aspects of the SDGs but, similarly to the SDGs, they are neither clearly included in national regulation nor nationally facilitated or enforced in relation to contaminated sediments. We propose that if management addresses the NEOs in ERA they might also in practice address several key aspects of the SDGs. By investigating the extent of the implementation of the environmental goals, such as the NEOs, we therefore have a unique opportunity to investigate which of the still new SDGs ought to be prioritized and how they are, or could be, implemented into ERA of contaminated sediments in Sweden.

## METHODS

The overall aim of the present study was to analyze the extent to which contaminated sediment ERAs in Sweden incorporate the SDGs and other environmental goals in their objectives and the assessment process. For example, are the goals included in the problem formulation, does the data collection enable assessment of potential effects to relevant goals, are risks affecting the goals described, and can resulting mitigation needs still be linked to relevant goals?

**Figure 1:**
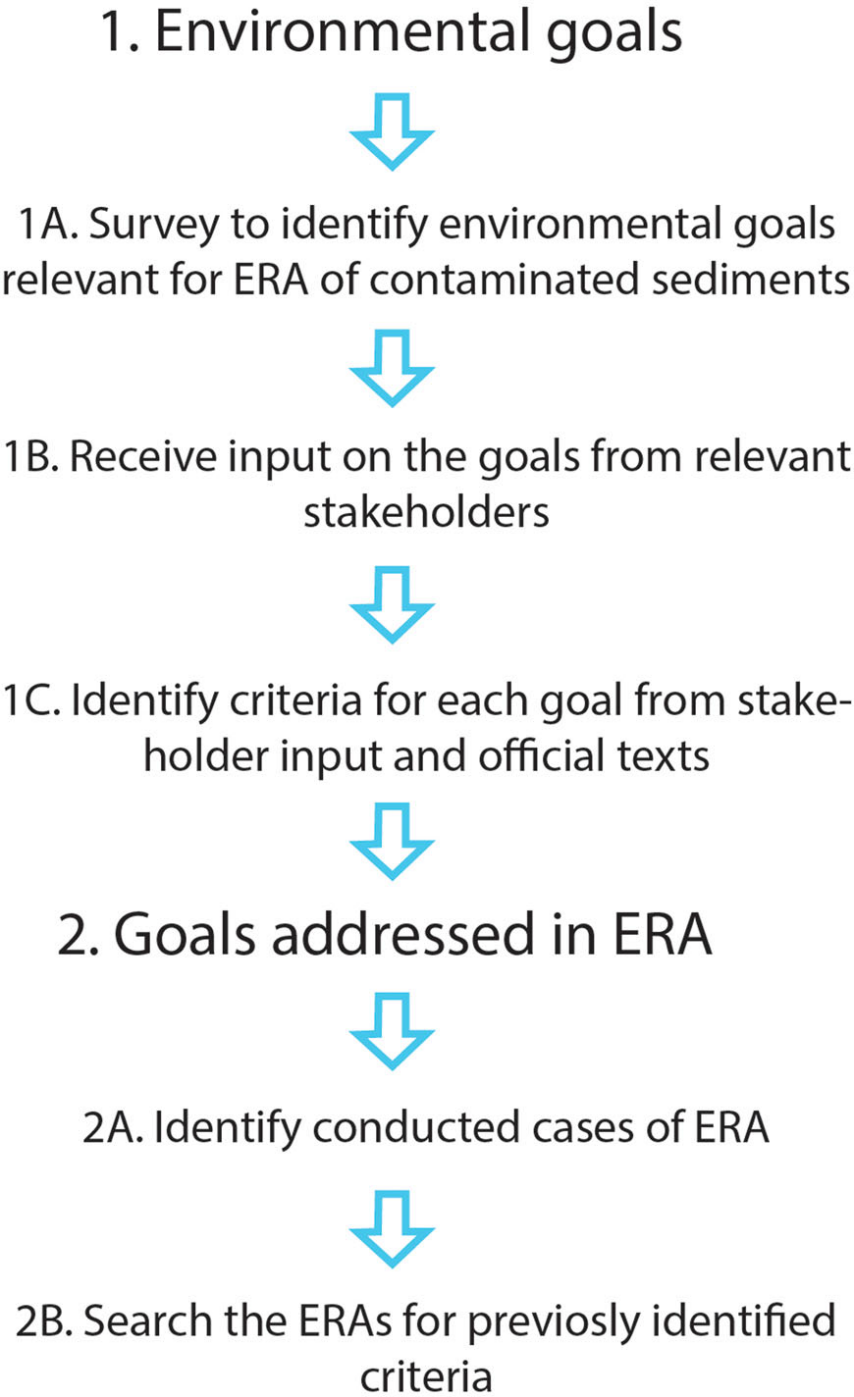
Study layout.

We conducted the study in steps where we: 1A) identified relevant goals through a survey sent to representatives working with contaminated sediment at governmental agencies, 1B) gained additional input on the goals through follow‐up interviews with some of the survey participants, 1C) identified criteria for each goal based on the goals' official texts and the interviews, 2A) identified ERAs of contaminated sediments sites conducted in Sweden, and 2B) searched for goals addressed in the ERA documents (Figure 1).

### Identifying goals

Not all environmental or development goals are necessarily generally applicable or as relevant or obvious as others are in relation to ERA of contaminated sediments (e.g., the SDG for “Affordable and clean energy,” which focuses on production of energy). To identify SDGs, NEOs, and other possible goals with links to contaminated sediments, or dependent on healthy sediments, we turned to a governmental agency network on contaminated sediments. It permanently involves representatives from 4 agencies that are closely involved in aspects of contaminated sediment, but from different perspectives. Together they cover management, guidance, policy and regulations, research, environmental monitoring, et cetera, related to contaminated sediments. The agencies were:

- the Swedish Environmental Protection Agency,
- the Swedish Geotechnical Institute,
- the Geological Survey of Sweden, and
- the Swedish Agency for Marine and Water Management.

Governmental agencies are obliged to work toward the implementation of the SDGs, and the work of these agencies relates specifically to several NEOs. The network organized a workshop in 2017 where, in addition to representatives from the member agencies, representatives were invited from 2 local administrative boards (Västra Götaland and Västernorrland), the City of Stockholm's administration board, and the Swedish Transport Administration. These 4 act as regulatory agencies and/or are responsible for sites with contaminated sediment. In order to identify goals relevant for sediment ERA, we invited all workshop participants to fill in an online survey before the workshop.

We designed the survey mainly according to a structured format based on the methodology described in Bryman (2008a, 2008b, 2008c). This format included standardized questions and a range of fixed‐choice answers followed by open‐ended questions. To reduce the workload for the respondents and to increase the response rate, we included a subset of 20 goals from the 16 NEOs (SEEPA 2012) and the 17 SDGs (UNDP 2018), with links to their official online descriptions. For each goal the respondents were asked to rate the relevance of the goal in relation to ERAs as “Not relevant,” “Partially relevant,” “Clearly relevant,” or “Unknown.” We also asked the respondents to elaborate on how they believed the goals were relevant. To identify additional goals, the survey ended with an open question asking for additional perspectives on which goals ERAs should address. For the open question we emphasized that additional goals perceived as relevant could be in the form of a directive, regulation, or similar and did not have to be one of the SDGs or NEOs.

### Input on the goals

We conducted semistructured follow‐up interviews with three of the respondents because it is recommended to confirm the results of such surveys (Alvesson 2010). We asked the interviewees to elaborate on how they perceived goals to be relevant for ERA and management. They gave their perspective on the goals they had marked as relevant as well as the additional goals suggested by other respondents in the survey. We also presented and discussed the survey results during the workshop with the representatives from the 8 agencies.

### Identify criteria for the goals

We considered it plausible that multiple environmental goals were addressed in ERA in Sweden given that the NEOs have been part of the authorities' work for quite some time (CEA 1999; SEEPA et al. 2018). However, because the general ERA guidance or regulations do not explicitly promote goals such as the SDGs, cases of ERA in Sweden might not have explicitly stated the intent to address any particular goal even though they did so in practice by addressing key aspects of the goals.

In order to determine if Swedish ERAs addressed the goals, we identified criteria for each goal that was described as relevant in the survey and follow‐up discussions. The goals' titles are based on clarifying texts with a number of targets, specifications, and indicators presented in their official descriptions. We used this information together with the comments on the survey, follow‐up discussions, and interviews to identify the criteria. When establishing the criteria we also identified keywords that were common in the texts describing the goals so that we could use them to screen ERAs for the criteria.

### Cases of ERA

Having identified the set of relevant goals and criteria, the next step was to investigate to what extent ERAs of contaminated sediment sites in Sweden met these criteria in practice. To find suitable reports, we sent a request to a contact list comprising staff at the regulatory agencies that supervise contaminated site management, and we contacted companies that conduct ERAs. We asked for reports that included ERAs of contaminated sediment sites.

### Identifying goals addressed in ERA

In order to identify if the reports addressed the relevant goals, we used content analysis, as described in Bryman (2008b) and Julien (2008), for example. This method can be used to identify explicit, latent, or otherwise underlying content through qualitative interpretation (Duriau et al. 2007).

We compared the ERA reports to the keywords and criteria and marked them manually using the software Nvivo 12 (QSR 2018). When searching the reports, we first searched and marked paragraphs with the goals' titles and keywords. We then systematically analyzed the paragraphs marked with keywords for each respective goal and compared the content to the previously identified criteria. For example, a paragraph describing an analysis of risks to human health by comparing concentrations of contaminants from sediment in fish to health guidelines was considered to meet the criteria of “stating an intent to investigate or reduce, risks from contaminated sediment to cause illnesses or deaths” connected to the goal “Good health and well‐being” (“Good health” for short). As another example, we considered paragraphs comparing results from measurements to the objectives stated in the Swedish regulation for aquatic management (SMEE 2004) to address the Water Framework Directive (WFD) (EU 2000). Whenever it was not clear if paragraphs addressed a goal, we further compared the paragraphs to the official descriptions of the goals identified as connecting them to contaminated sediment.

To investigate if the ERAs addressed the goals continuously throughout the whole ERA process, we also noted which goals were addressed in which of the phases of ERA, namely the problem formulation, analysis, and risk characterization phases. To add resolution, we also took separate note of 2 parts in the risk characterization: first, the part describing the effects and potential effects based on the results from individual measurements or lines of evidence conducted in the analysis, and second, the integration of the results into concluding recommendations for the need of risk reduction. Most of the ERAs were clearly divided into these phases. For those that were not, the classification was based on whether the content described the background and objectives, described the methods used to assess risk, characterized the risks, or described the reduction needs in relation to contaminated sediments.

## RESULTS

### Relevant goals

The survey provided us with a set of goals considered directly relevant for ERAs of contaminated sediment, by people who are key to management of contaminated sediment at all 8 agencies addressed. The Swedish Agency for Marine and Water Management took the opportunity to discuss the goals internally and answered as a group, whereas the personnel from other agencies answered individually, totaling 12 responses.

A majority of the respondents ranked 5 goals as directly relevant (Table 1); the first 3 goals are SDGs, and the remaining are NEOs:

- Clean water and sanitation (“Clean water” for short)
- Life below water (“Life in water”)
- Life on land (“Life on land”)
- Flourishing lakes and streams (“Flourishing lakes”)
- A balanced marine environment, flourishing coastal areas and archipelagos (“Marine balance”).

**Table 1:** Goals for environmental management in relation to environmental risk assessment, graded by 12 responses in a survey addressed to staff at Swedish regulatory and advisory authorities

Environmental goals	Condensation of goal titles	Directly relevant	Partially relevant	Not relevant	Unknown
A balanced marine environment, flourishing coastal areas and archipelagos^b^, ^d^	Marine balance	12	0	0	0
Life below water^a^, ^d^	Life in water	11	1	0	0
Flourishing lakes and streams^b^, ^c^	Flourishing lakes	11	1	0	0
Clean water and sanitation^a^, ^c^	Clean water	10	1	1	0
Life on land^a^, ^c^	Life on land	9	2	1	0
Good health and well‐being^a^	Good health	6	5	0	1
A rich diversity of plant and animal life^b^	Biodiversity	6	4	1	0
Sustainable cities and communities^a^	Sustainable cities	4	6	2	0
Good‐quality groundwater^b^	Quality groundwater	4	5	3	0
Reduce climate impact^b^	Climate impact	4	5	3	0

^a^ Sustainable development goals (UN 2015).

^b^ Swedish National Environment Objectives (SEEPA 2012).

^c^ Goal relevant only for freshwater systems.

^d^ Goal relevant only for marine systems.

The goals all explicitly target healthy ecosystems to some extent, and all but “Life on land” explicitly address the need to manage pollution in the aquatic environment. “Clean water” targets human consumption, that is, focuses on human health but includes healthy ecosystems as a prerequisite. The other 4 goals relate directly to healthy ecosystems and target healthy aquatic environments in marine or inland freshwater ecosystems, both for their intrinsic value and as a resource for society.

Most of the goals perceived as relevant address sustainable management of the ecosystem (Table 2), that is, the goals clearly overlap in that respect. In addition to the 5 goals perceived to be most relevant, sustainable management is also an explicit part of “A rich diversity of plant and animal life” (“Biodiversity” for short) and the WFD. These goals all explicitly target protection or improvement of ecosystems.

**Table 2:** Analytical framework with criteria used to connect paragraphs from the analyzed ERAs to environmental goals

Goals	Criteria: Content stating an intent to investigate or reduce risks from contaminated sediment to each goal^a^	Goal clarification used as the basis for the criteria^b^
1 Good health	Cause illnesses or deaths.	3.4. and 3.9: By 2030, substantially reduce the number of deaths and illnesses from hazardous chemicals and air, water, and soil pollution and contamination.
2 Clean water	Drinking water quality and ecosystems important for that quality.	6.3. By 2030, improve water quality by reducing pollution, eliminating dumping, and minimizing release of hazardous chemicals and materials.
		In addition, 6.6.
3 Life in water	Cause contamination or negative consequences to marine and coastal ecosystems.	14.1. and 14.2. By 2020, sustainably manage and protect marine and coastal ecosystems to avoid significant adverse impacts, including by strengthening their resilience, and take action for their restoration in order to achieve healthy and productive oceans.
4 Life on land	Maintenance and restoration of ecosystems and ecosystem services.	15.1. By 2020, ensure the conservation, restoration, and sustainable use of terrestrial and inland freshwater ecosystems and their services, in particular forests, wetlands, mountains, and drylands, in line with obligations under international agreements.
	Cause destruction of natural habitats and loss of biodiversity and species.	In addition, 15.5.
5 Flourishing lakes	At least good ecological status in accordance with Regulation 2004:660 (SMEE 2004).	1. Lakes and streams have at least a good ecological status or potential and good chemical status in accordance with the regulation (SMEE 2004) on the quality of the aquatic environment.
	Ecosystem services and the recovery of threatened species and habitats in valuable freshwater locations.	In addition, 4, 7, and 10.
6 Marine balance	Maintenance of sustainable biological production and biodiversity and ecosystem services.	1. Coastal and marine waters will have a good environmental status in regard to physical, chemical, and biological parameters in accordance with the regulation for the marine environment (2010:1341) (SMEE 2010).
	Good environmental status in accordance with the Swedish Regulation 2010:1341 (SMEE 2010).	In addition, 2, 3, 4, 6, and 10.
	At least good ecological status or potential and good chemical status in accordance with the Swedish regulation 2004:660 (SMEE 2004).	
	Recovery of threatened species and habitats in valuable locations.	
7 Biodiversity	Natural habitats and ecosystems and their functions and processes.	3. The ecosystems have the ability to resist disturbance and adapt to change, such as a changing climate, so that they can continue to deliver ecosystem services and contribute to counteracting climate change and its effects.
	Public access to a good natural and cultural environment.	
	Resilience of ecosystems.	
8 No toxins	Threaten human health or biodiversity as part of the total exposure from all contaminants in the environment.	1. The combined exposure to chemical substances via all exposure pathways is not harmful to humans or biodiversity.
	Be above background levels, for naturally occurring substances, or above zero for synthetic substances.	In addition, 4, 5.
9 WFD	Protection or restoration of ecosystems to an at least good status in accordance with the Swedish regulation for water management (2004:660) (SMEE 2004).	The directive sets out rules to halt deterioration in water bodies and achieve “good status” for lakes and groundwater, dictated by the Swedish regulation for water management (2004:660) (SMEE 2004).
10 Sustainable cities	Access for all to safe housing.	11.4. Strengthen efforts to protect and safeguard the world's cultural and natural heritage.
	Protection of cultural and environmental heritages.	
11 Quality groundwater	Quality of groundwater, which has to be at least “good,” in accordance with the regulation of management of the aquatic environment (2004:660) (SMEE 2004).	2. Groundwater covered by the regulation of management of the aquatic environment (2004:660) (SMEE 2004) has a good chemical status.
12 Climate impact	Reduction of greenhouse gas concentrations in the atmosphere.	1. No net release of greenhouse gases by 2045.

ERA = environmental risk assessment; WFD = Water Framework Directive.

^a^ We used several specific clarifications, that is, targets and specifications, to create the criteria.

^b^ For each goal, a clarification is written out as an example. In the official texts, the clarifications are numbered, and the numbers for those used are provided in the table. The clarifications can be found at: http://www.sverigesmiljomal.se (only in Swedish), http://www.undp.org/content/undp/en/home/sustainable-development-goals.html, and https://eur-lex.europa.eu/legal-content/EN/TXT/?uri=LEGISSUM:l28002b.

The remaining goals also address sustainable management of the environment but focus on aspects other than the ecological. “Good health and well‐being” (“Good health” for short), “A nontoxic environment” (“No toxins”), and “Good‐quality groundwater” (“Quality groundwater”) target human health; “Sustainable cities and communities” (“Sustainable cities”) targets society, whereas “Reduced climate impact” (“Climate impact”) targets the reduction of greenhouse gas emissions (Table 1).

Several of the goals identified as relevant in the survey explicitly address either marine or freshwater systems. Hence, in the following analysis of Swedish ERAs, we did not search reports for goals that were not relevant for their respective sediment system. This meant that we searched the reports that address a marine sediment system for fewer goals than those that address freshwater systems. For example, the clarification of the goal “Life in water” explicitly refers to marine and coastal environments (see Table 2).

### Input on the goals

Based on propositions from the survey and follow‐up interviews, we added 2 goals, in addition to the 10 most relevant goals, to the following analysis. We added the NEO “No toxins” and the EU WFD (EU 2000). The “No toxins” NEO explicitly targets reduced environmental pollution, and the EU WFD is implemented through the Swedish Environmental Code (SMEE 1998) and regulations for water management (SMEE 2004, 2010). We included the WFD as a goal for sustainability because, as an EU directive, it sets goals for the aquatic environment and dictates laws and regulations in order to reach that goal. The link from the WFD and “No toxins” to contaminated sediments is intrinsic, given that they are already the pillars upon which relevant frameworks should be based.

### Criteria for the goals

We identified the criteria to use in the content analysis from the official Swedish descriptions of the goals, using the input from the communication with the participants to the follow‐up interviews and comments on the survey. For the NEOs, we derived the criteria from the specifications for each goal that clarify what aims the overall objective should meet. Similarly, each SDG has a number of targets describing the goals in detail. The WFD is an EU directive, but its implementation is based on national adaptations. In Sweden, the national Agency for Marine and Water Management manages the WFD; therefore, even though the original directive is the basis for the WFD, the criteria we identified are based on regulations specific for the WFD in Sweden. Table 2 depicts each goal and corresponding criterion with examples of the basis for the criterion. For the majority of the relevant goals, several targets or specifications were clearly relevant for contaminated sediments; in Table 2 we included 1 example per goal. We conducted the search of latent content addressing the goals by first searching for the keywords (Table 3). For each goal we then compared the paragraphs marked with the goal's respective keywords to the criteria, and when unclear, to the original clarifications of the goal connected to contaminated sediment. Through this process, the material was analyzed carefully and thoroughly; most paragraphs were marked with several keywords for several goals and were read iteratively.

**Table 3:** List of keywords used to screen for content that potentially met criteria for the environmental goals relevant to address in ERA of contaminated sediments

Goals^a^	Keywords
5, 6, 9	Chemical status
1, 2, 8	Pollution, Chemicals, Environmental contaminant
1, 4, 7, 8	Food web, Food chain, Foodstuff, Biomagnification
1, 8	Exposure, Exposure pathways, Spread
2, 6, 9	Water quality
6, 9	Environmental status
1, 2, 11	Drinking water
4, 5	Endangered species
3, 4, 7, 8, 6	Biodiversity
5, 6, 10	Cultural environment, Historic cultural remnants
2, 3, 4, 5, 6, 7, 9	Ecosystem, Ecological status, Ecology
3, 4, 5, 6, 7	Ecosystem services, Production capability, Blue growth, Aquaculture, Resource
2, 3, 4	Restoration
4, 5, 6	Habitat, Green infrastructure
4, 7	Disturbance, Nesting, Benthic fauna
3, 4	Preservation, Protection
8	Limit values, Background levels, Values, Concentrations, Thresholds
9	Surface water
10	Exploitation, City
11	Groundwater
12	Climate, Emissions, Greenhouse gas, Temperature, Energy

ERA = environmental risk assessment.

^a^ The goals are numbered as follows: 1) Good health and well‐being; 2) Clean water and sanitation; 3) Life below water; 4) Life on land; 5) Flourishing lakes and streams; 6) A balanced marine environment, flourishing coastal areas and archipelagos; 7) A rich diversity of plant and animal life; 8) A nontoxic environment; 9) Water Framework Directive; 10) Sustainable cities and communities; 11) Good‐quality groundwater; 12) Reduced climate impact.

### Cases of ERA

In response to our request for ERAs of contaminated sediment sites, we received a variety of documents. We selected 7 reports for the present study. All of the reports were written between the years of 2008 and 2015 and presented completed ERAs to regulating agencies. They described the following phases of ERA:

- Problem formulation—including the assessment's background and objectives.
- Analysis—including measurements, analysis, and results.
- Risk characterization—including characterization of effects and potential effects, concluding integration of risks and reduction needs.

Two ERAs were conducted for a private company and 5 for municipalities that had assumed responsibility for cases where the original polluter was no longer held accountable. One report addressed a marine system and the others freshwater systems (Table 4). We analyzed the entirety of the reports except for parts that explicitly addressed only terrestrial areas or groundwater without connection to sediments.

**Table 4:** Cases of ERA of contaminated sediments, analyzed in order to investigate how Swedish ERA addresses goals for a sustainable environment

	Cases of ERA	Conducted for
A	ERA of multiple types of contaminants in a marine harbor.	Municipality
B	ERA of multiple types of contamination in an inland bay, from a paper mill.	Municipality
C	ERA of organic contaminants from oil spills in a lake, from industry.	Private company
D	ERA of organic contaminants from oil spills in a lake, from industry.	Private company
E	ERA of Hg contamination from wood pulp industry, in an inland bay.	Municipality
F	ERA of pyrite ash contamination from pyrite production, in a stream.	Municipality
G	ERA of organic contaminants from industrial impregnation, in an inland bay.	Municipality

ERA = environmental risk assessment.

### Goals addressed in ERA

Two reports referred to a number of goals explicitly. Report A addressed the NEOs “Marine balance” and “No toxins” and the WFD (Table 5). The goals were not explicitly part of the planning, analysis, or assessment of results, but were referred to when describing the need to mitigate the risks in the concluding recommendations for risk reduction. The second report, G, explicitly addressed the WFD and used the chemical objectives stipulated in the WFD as part of the analysis and characterization of risk, using the WFD objectives as a point of reference to determine the level of risk.

**Table 5:** The ERAs analyzed in this study (A–G), with the individual environmental goals they addressed in total and per respective part of the assessment^a^

ERA	Goals addressed in total per ERA		Problem formulation	Analysis	Risk characterization: description of risk	Risk characterization: concluding needs for reduction
A	7	Good health	Good health	Good health	Good health	Life in water
		WFD	Life in water	Marine balance	WFD	Marine balance^b^
		Life in water	Marine balance	No toxins	Life in water	No toxins^b^
		Marine balance	Biodiversity	Sustainable cities	Marine balance	WFD^b^
		Biodiversity	No toxins		Biodiversity	
		No toxins			No toxins	
		Sustainable cities				
B	4	Good health	Good health	Good health	Good health	No toxins
		Life on land	Life on land	No toxins	Clean water	
		Biodiversity	Biodiversity		No toxins	
		No toxins	No toxins			
C	4	Good health	Good health	Good health	Good health	No toxins
		Life on land	Life on land	Life on land	Life on land	
		Biodiversity	Biodiversity	Biodiversity	Biodiversity	
		No toxins	No toxins	No toxins	No toxins	
D	3	Life on land	No toxins	No toxins	Life on land	Life on land
		Biodiversity			Biodiversity	Biodiversity
		No toxins			No toxins	No toxins
E	5	Good health	Good health	Good health	Good health	Life on land
		Clean water	Life on land	Clean water	Clean water	Biodiversity
		Life on land	Biodiversity	Life on land	Life on land	No toxins
		Biodiversity	No toxins	Biodiversity	Biodiversity	
		No toxins		No toxins	No toxins	
F	5	Good health	Good health	Good health	Good health	Life on land
		Clean water	Clean water	Clean water	Clean water	Biodiversity
		Life on land	No toxins	Life on land	Life on land	No toxins
		Biodiversity		Biodiversity	Biodiversity	
		No toxins		No toxins	No toxins	
G	7	Good health	No toxins	Life on land	Good health	Life on land
		WFD		Flourishing lakes	WFD	Biodiversity
		Clean water		Biodiversity	Clean water	No toxins
		Life on land		No toxins	Life on land	
		Flourishing lakes		WFD^b^	Flourishing lakes	
		Biodiversity			Biodiversity	
		No toxins			No toxins	

ERA = environmental risk assessment; WFD = Water Framework Directive.

^a^ Goal titles are shortened; see Table 1 for their full names.

^b^ Goals were addressed explicitly.

According to the analysis using the criteria, all ERAs addressed risks to the environment or human health from chemicals released by industrial activities in all phases and thus met the criteria for the NEO “No toxins,” making that goal the most frequently addressed and highlighting its ubiquity in Swedish ERA.

The 5 goals highlighted as most relevant in the survey were addressed to a varying degree (Tables 1 and 6). Part of the explanation for this is that the goals considered most relevant are exclusive to either marine or freshwater systems. Only Report A assessed a marine system, and it met the criteria for the 2 goals exclusive to marine systems, “Life in water” and “Marine balance” (Table 5). Report A did not address the 3 other most relevant goals, which were explicit for freshwater systems, “Life on land,” “Clean water,” and “Flourishing lakes.”

**Table 6:** The frequency at which analyzed ERAs met criteria for the environmental goals in 4 phases of assessment^a^

Goals	Nr ERAs	Problem formulation	Analysis	Risk characterization: description of risk	Risk characterization: reduction needs
No toxins^c^	7	7	7	7	7
Good health^b^	7	5	5	6	0
Biodiversity^c^	7	4	4	6	4
WFD^d^	7	0	1	2	1
Sustainable cities^b^	7	0	1	0	0
Quality groundwater^c^	7	0	0	0	0
Climate impact^c^	7	0	0	0	0
Life on land^b^	6	3	4	5	4
Flourishing lakes^c^	6	0	1	1	0
Clean water^b^	6	1	2	4	0
Marine balance^c^	1	1	1	1	1
Life in water^b^	1	1	0	1	1

ERA = environmental risk assessment; WFD = Water Framework Directive.

^a^ The first 7 goals were relevant for all 7 cases of ERA; Life on land, Flourishing lakes, and Clean water were specific for ERAs that address freshwater sediments; and Marine Balance and Life in water were relevant for the ERA that addressed marine sediments.

^b^ Sustainable development goals (UN 2015).

^c^ Swedish national environment objectives (SEEPA 2012).

^d^ Directive from the European commission (EU 2000).

All the ERAs that addressed freshwater systems (B–G) met the criteria for “Life on land.” They did so frequently in all phases, especially in risk characterization, describing effects or risks of effects to the ecosystem. “Clean water” and “Flourishing lakes” were addressed to a more variable degree. Most ERAs (B, E, F, G) addressed “Clean water” (Tables 5 and 6) by assessing drinking water in relation to contaminated sediment. In the same way as for “Life on land,” the ERAs addressed “Clean water” mainly when describing effects and potential effects and less frequently in other parts. Only 1 report, G, met the criteria for the goal “Flourishing lakes” by assessing the chemical status in the water according to the regulations stipulated in the goal.

All ERAs also addressed two or more of the goals related to managing ecosystems (i.e., “Clean water,” “Life in water,” “Life on land,” “Flourishing lakes,” “Marine balance,” “Biodiversity,” and the WFD) but not in all phases of assessment. The most commonly addressed goals with an explicit focus on the ecosystems were “Life on land” and “Biodiversity” (Table 6). Although all ERAs addressed ecosystem management, for example, by connecting pollution to ecosystem effects, none of them addressed ecosystem services or the environment as a resource, which is an aspect the 5 most relevant goals have in common.

In addition to the aspects of ecology and pollution, all ERAs frequently addressed the aspect of human health. Looking at the first 3 assessment phases, the criteria for the goal “Good health” were fulfilled more frequently than criteria for any goals focusing on managing the ecosystem (Table 6). All of the ERAs specifically related the sediment contamination to potential human health effects. Most ERAs did so in all of the phases of ERA, up to and including the risk characterization. However, in the risk characterization none of the ERAs concluded with any recommendations for risk reduction regarding human exposure or health effects.

The goals most frequently addressed by the ERAs after “No toxins” were “Biodiversity,” “Good health,” and “Life on land,” in decreasing order of frequency (Table 6). Among the 10 most relevant goals, the fewest respondents considered “Sustainable cities,” “Quality groundwater,” or “Climate impact” relevant. The ERAs reflect this; “Sustainable cities” is addressed only in the analysis phase of Report A, and “Quality groundwater” and “Climate impact” by none of the ERAs (Table 5).

## DISCUSSION

In the present study, all the ERAs explicitly addressed human health (Table 6). In some cases this might be highly relevant, but often the risks to ecological receptors are greater than to human health, according to some of the available literature on sediment ERA. Some approaches actually omit the human health aspect to focus mainly on biological or chemical aspects (e.g., Chapman and Hollert 2006; Scrimshaw et al. 2007; Anderson et al. 2008). The analyzed ERAs all concluded that, in their cases, the risks to human health were negligible, even in the cases where the levels of contaminants were substantially elevated.

The ERAs' conclusions and recommendations for risk reduction again indicate a large influence from the ERA framework for soil where the NEO “No toxins” is considered central. Due to this influence, there is a risk that assessors do not recognize that ecological risks could be expected to be more pronounced for contaminated sediments compared to soil. Goals with an explicit focus on ecosystem effects are addressed at a lower frequency than “No toxins” (Table 6). This is in contrast to the survey, which indicates that a broader range of values should be addressed in Swedish ERA of contaminated sediments.

The sediment environment is often more dynamic and difficult to predict than is the soil environment. The movements of the sediments en masse or of contaminants between sediment, water, and biota often require extensive understanding and appropriate tools to successfully manage contamination and address goals. Dealing with contaminated sediments is also complex from a regulatory perspective. However, it is difficult for assessors, and to some extent even for authorities, to get an overview of all regulations that are, or might be, relevant when managing contaminated sediments (Severin et al. 2018). Nor are there any standard directives for how to implement such regulations, for example, the WFD (EU 2000), when managing contaminated sediments as a contaminated site. As described above in the *Background* section there is a lack of facilitation in contaminated sediment management in Sweden. However, it is argued that facilitation in tandem with regulation and enforcement are important tools to promote implementation of top–down decisions, such as the implementation of the SDGs, into management (Victor et al. 1998; Weiss and Jacobson 1998).

Of the goals identified as relevant in the present study, only “Flourishing lakes” and “Marine balance” directly refer to regulations, and the WFD is in itself a regulation (EU 2000; SMEE 2004, 2010). However, the connection to regulation does not seem to have had a strong influence because those goals are among the least addressed by the ERAs. On the contrary, the most frequently addressed goals, “No toxins” and “Life on land,” do not explicitly refer to any regulations, nor are they mentioned in any regulations for sediment or contamination. A clearer connection between ERA and regulation could possibly improve implementation into management. However, even with regulation, stakeholders will still need support and facilitation from the authorities. Such facilitation might improve over the coming years, given that there appears to be a new focus and increased funding from the Swedish government targeted at restoration of the aquatic environment since 2018 (Severin et al. 2018).

It might be suspected that the lack of regulation and guidance directly relating management to many of the goals might make private stakeholders less motivated to address the goals compared to public stakeholders that are obliged to work toward the goals. Of the ERAs included in the present study, those that addressed the greatest number of goals were indeed conducted for public stakeholders. This suggests that regulation, facilitation, and enforcement are especially important to promote the goals to the private sector. However, the number of analyzed ERAs is low, and there were ERAs funded by public means among those that included the lowest number of goals. With the currently increased efforts in managing contaminated sediment sites, it will be interesting to see if this observation is a trend, and if so, how new guidance manages to handle it. It is an open question whether it is possible to regulate and enforce to the extent that each management action effectively supports all relevant long‐term environmental sustainability goals.

None of the goals highlighted in the survey related directly to adaptation to climate change effects, and the majority of respondents did not consider the goal “Climate impact” or other goals focusing on climate change as clearly relevant (Table 3). Likewise, none of the ERAs made connections or references to the goal “Climate impact.” As part of the ERA analysis, we searched for keywords related to climate change in general but found none in any of the ERAs, indicating that climate change is not taken into consideration. Because adaptation to climate change was not part of any of the identified goals, we did not pursue the issue further. However, the implications for risk from a changing climate is likely to become more pronounced and is often missing from ERAs. Chapman (2012) argued that ERA strategies and frameworks often fail to account for long‐term requirements related to climate change. This increases the uncertainties long term when assessing risks from contaminants (Landis et al. 2013).

## CONCLUSIONS

In conclusion, several SDGs and NEOs are directly relevant to ERA of contaminated sediments, and ERAs and subsequent remediation actions could potentially contribute more to fulfilling several of the relevant goals than they do today. However, current guidance for contaminated sites in Sweden lacks detail on managing contaminated sediments, and it is unclear for stakeholders how to relate goals adopted by the state. Existing ERA guidelines in Sweden do not specifically address contaminated sediments, nor is the connection clear for ERA in relation to regulations for water management, such as the WFD. There is also a lack of support on how to address the identified set of goals in relation to contaminated sediments in general. We argue that this is not in line with Sweden's outspoken intent to be leaders in sustainable management in general and in the work toward the SDGs in particular.

We show a discrepancy between the goals that authorities consider most relevant and those that are addressed in the assessments. The explanations for this are likely in part the lack of facilitation and directives for assessors and different objectives for relevant authorities. However, the lack of facilitation has been recognized and efforts to cooperate between authorities have increased (Severin et al. 2018), so the abovementioned difficulties could change in the coming years.

Personnel at governmental agencies working with contaminated sediments in Sweden stress the importance of ecological values and the capability and sustainable use of the environment. In contrast to this, ERAs might be overly affected by experiences from assessing contaminated soils, not giving due focus to ecological aspects that are key to a majority of the goals relevant for sediment management.

The present study suggests that guidance on how to assess contaminated sediments needs to address a broader range of environmental aspects than traditional ERA for soils in Sweden. The connection between the goals and ERAs should be made clearer in regulation and in a future guidance for ERA. It would be valuable to develop a system or tool to help keep attention on the goals or to check their relevance at all phases of the ERA process. One way to incorporate the identified goals in ERA guidance could be to provide a set of assessment and measurement endpoints linked to the goals, for example, based on the criteria identified in Table 2.

Overall, the identified set of goals could readily be incorporated into Swedish ERA methodologies. Even though there is no guidance that requests assessors to address environmental goals today, all investigations included in the present study, at least indirectly, address several of the SDGs and NEOs. In addition, several ecological factors were addressed, at least in general terms, in the early stages of the ERA and thereby criteria for multiple SDGs. This suggests a readiness to include the ecological dimension that could pave the way when tools and guidance are in place. If the guidance includes and promotes the different “ecological” SDGs and provides guidance on how to address them, there should be a good potential for sustainable management and fulfillment of several relevant parts of the SDGs.

In Sweden, it is entirely possible that support for SDGs in ERA of contaminated sediments can be improved. The Swedish environmental code is based upon sustainable development principles, the country is committed to being a leader toward the SDGs, and relevant authorities experienced in working toward environmental goals have recently established increased cooperation on contaminated sediments. Sweden should be well equipped to develop the management framework for contaminated sediments so that it supports the implementation of the SDGs and ensures sustainable management.

## SUPPLEMENTAL DATA

Survey questionnaire

Survey results: Closed questions

Survey results: Open questions

Follow up‐interview questionnaire

**Interview 1.** Questionnaire and answers

**Interview 2.** Questionnaire and answers

**Interview 3.** Questionnaire and answers

## Supporting information

This article contains online‐only Supplemental Data.

Supporting information

## Data Availability

The data produced from this study and material used are made available in the supplemental data. Material used for this study that can be used to identify individuals will not be made public to corresponding author Peter Bruce (peter.bruce@su.se).
