# Supplementary material for: Environmental Goals Addressed in Assessments of Contaminated Sediments
Source: Integr Environ Assess Manag. 2019 Dec 19;16(1):128–39. doi: 10.1002/ieam.4223 (PMC6973226; doi:10.1002/ieam.4223)
Supplement: Supplementary file 1 — Supporting information [file IEAM-16-128-s001.docx]

# Supplemental Data to the manuscript - Environmental goals addressed in assessments of contaminated sediments

## Content

- Survey questionnaire
- Survey results – closed questions
- Survey results –open questions
- Follow up interview Questionnaire
  - Interview 1 – questionnaire and answers
  - Interview 2 - questionnaire and answers
  - Interview 3 - questionnaire and answers

## Survey

The following description is translated from the online survey conducted for the manuscript – Environmental goals addressed in assessments of contaminated sediments, originally in Swedish.

### Introduction

The aim of this study it to investigate how our societal goals for sustainability and environment are reflected in our management of contaminated sediment. Through this survey, we will establish a list of goals that the concerned stakeholders perceive to have bearing on the management of contaminated marine and freshwater sediments.

### Layout

The respondents were encouraged to answer three questions for each of the goals listed below. For each goal, links were provided to the official online descriptions. Lastly, the respondents were also encouraged to suggest additional goals they perceived as relevant in relation to contaminated sediments.

1. Is the goal relevant for management of contaminated sediments? Choose an answer among either: Directly relevant, Partially relevant, Not relevant, Do not know. Multiple choice question.
2. For the goals that are considered relevant, is it either: Directly, or Indirectly relevant? Multiple choice question.
3. Motivate how the relevant goals connect to management of contaminated sediment. Open question.

| - Life below Water |
| --- |
| - A Balanced Marine Environment, Flourishing Coastal Areas and Archipelagos |
| - Clean Water and Sanitation |
| - Flourishing Lakes and Streams |
| - Good health and Well-being |
| - Life on Land |
| - A Rich Diversity of Plant and Animal Life |
| - Sustainable Cities and Communities |
| - Good-Quality Groundwater |
| - Reduce Climate Impact |
| - Industry, Innovation and Infrastructure |
| - Thriving Wetlands |
| - Good Built Environment |
| - Decent Work and Economic Growth |
| - Climate action |
| - Zero Eutrophication |
| - Clean Air |
| - Natural Acidification Only |
| - A Safe Radiation Environment |
| - A Varied Agricultural Landscape |

## Survey results – closed questions

Table 1. The ranked relevance of 20 goals identified in 12 survey responses. The respondents choose answers from “Directly relevant”, “Partially relevant”, “Not relevant” or “Unknown”. The list is ordered in a falling order of relevance with the goals perceived to be relevant for most respondents at the top. The topmost ten were included in the analysis for the study.

| **Environmental goals** | **Directly relevant** | **Partially relevant** | **Not relevant** | **Unknown** |
| --- | --- | --- | --- | --- |
| Life below Water | 11 | 1 | 0 | 0 |
| A Balanced Marine Environment, Flourishing Coastal Areas and Archipelagos | 12 | 0 | 0 | 0 |
| Clean Water and Sanitation | 10 | 1 | 1 | 0 |
| Flourishing Lakes and Streams | 11 | 1 | 0 | 0 |
| Good health and Well-being | 6 | 5 | 0 | 1 |
| Life on Land | 9 | 2 | 1 | 0 |
| A Rich Diversity of Plant and Animal Life | 6 | 4 | 1 | 0 |
| Sustainable Cities and Communities | 4 | 6 | 2 | 0 |
| Good-Quality Groundwater | 4 | 5 | 3 | 0 |
| Reduce Climate Impact | 4 | 5 | 3 | 0 |
| Industry, Innovation and Infrastructure | 3 | 8 | 0 | 1 |
| Thriving Wetlands | 4 | 2 | 5 | 1 |
| Good Built Environment | 1 | 8 | 3 | 0 |
| Decent Work and Economic Growth | 2 | 5 | 5 | 0 |
| Climate action | 2 | 6 | 3 | 1 |
| Zero Eutrophication | 1 | 7 | 3 | 1 |
| Clean Air | 2 | 2 | 6 | 2 |
| Natural Acidification Only | 1 | 3 | 7 | 1 |
| A Safe Radiation Environment | 0 | 1 | 10 | 1 |
| A Varied Agricultural Landscape | 1 | 5 | 4 | 1 |

## Survey results –open questions

Table 2. List of 10 additional goals suggested to be directly relevant by a minority of the respondents as a response to an open question in the survey on which societal goals for a sustainable environmental management of contaminated sediments. The two goals “A non-toxic environment” and the “Water framework directive” were described as intrinsic to management of contaminated sediments in the follow up interviews. They were therefore included in the analysis for this study

| Marine environment framework ^1^ | No Poverty ^3^ |
| --- | --- |
| Water framework directive ^1^ | Quality Education ^3^ |
| Bathing water directive ^1^ | Gender Equality ^3^ |
| Baltic Sea Action Plan ^2^ | A Non-Toxic Environment ^4^ |
| Zero Hunger ^3^ | EU Strategy for the Baltic Sea Region |

1 EU directive, 2 HELCOM strategy, 3 UN Sustainable Development Goal, 4 Swedish environmental objective.

## Follow up interview Questionnaire

The information below is translated from the questionnaires and interview notes originally in Swedish. The full transcripts are only available in Swedish.

**Description of the aim of the interview**

The following text in Swedish was submitted to the interviewee and discussed prior to the interview:

The aim is to investigate which of the society’s goals for sustainability that are affected by the management of contaminated sea and freshwater sediments. The interview is based on the answers the interviewee gave to an earlier survey on the goals for management of contaminated sediment. The questions are meant to investigate in which way the goals pointed out, by the interviewee, to be relevant are relevant for management of contaminated sediments.

**Layout**

First the interviewee is encouraged to describe how she experiences that contaminated sediments affect the goals she pointed out as directly relevant, followed by the goals pointed out as partially relevant. Finally, the interviewee and other respondents suggested additional goals in the survey. The Interviewee is asked to describe the connection she sees between those and management of contaminated sediment. The first two parts only concerns UN Sustainable Development Goals and Swedish National Environmental Objectives. The last part addressed additional forms of environmental goals the survey respondents perceived to be relevant for contaminated sediments.

- 1. What is the connection between the directly relevant goals and management of contaminated sediments?
  2. What is the connection between the partially relevant goals and management of contaminated sediments?
  3. Does the interviewee agree to the notion that the additional goals suggested by other respondents to the survey are relevant to management of contaminated sediments? If so, what connection do the interviewee see?

### Questionnaire and answers from interview 1.

#### Part 1. What is the connection between the directly relevant goals and management of contaminated sediments?

Notes from part 1 of the follow up interviews regarding the goals indicated as directly or partially relevant by the respondents. Each interviewee had earlier indicated whether the relation between each goal in the table to contaminated sediments was one of the following: Directly relevant, Partially relevant, Not relevant, or Unknown. The goals in part 1 are all either UN Sustainable development goals or Swedish National Environmental Objectives.

| ***Goal - Good health and well-being*** |
| --- |
| Partially relevant. Due to effects on health connected to food consumption. |
| ***Goal -* Clean water and sanitation** |
| Interviewee 1: Directly relevant. Contaminated sediments in relation to drinking water. |
| ***Goal -* Life below water** |
| Directly relevant. Contaminated sediments in relation to aquaculture, drinking water. |
| ***Goal -* Life on land** |
| Interviewee 1. Directly relevant. Contaminated sediments affect biodiversity. |
| ***Goal -* Flourishing lakes and streams** |
| Directly relevant. Sediments are integral to the objective. |
| ***Goal -* A balanced marine environment, flourishing coastal areas and archipelagos** |
| Directly relevant. Connecting to contaminated sediments through exploitation and boat traffic, those activities should not negatively affect the water. |
| ***Goal -* A rich diversity of plant and animal life** |
| Directly relevant. Effects on organisms during management of contaminated sediments. |
| ***Goal -* Decent work and economic growth** |
| Directly relevant. Economic development through blue growth and the effects on economic opportunities by management of contaminated sediments. |
| ***Goal -* Good-quality ground water** |
| Directly relevant. Clear connection when water move from lakes to groundwater. |
| ***Goal -* Reduced climate impact** |
| Partially relevant. Temperature and the effects on sediment fluxes. Increased rainfall and currents. |
| ***Goal -* Sustainable cities and communities** |
| Directly relevant. Relevant in relation to exploitation. |
| ***Goal -* Thriving wetlands** |
| Directly relevant. Risk of water contaminated from sediments to reach wetlands during flooding events. |
| ***Goal -* Good built environment** |
| Partially relevant. Living and recreation in relations to contaminated sediments. Extra important for our agency. |

#### Part 2. What is the connection between the partially relevant goals and management of contaminated sediments?

The respondent is asked to explain the connection between contaminated sediment and additional goals the respondent suggested were relevant when answering the survey.

***Goal -* Marine environment directive**

Explanation: Directly relevant in many aspects. Addresses the integrity of the bottom.

***Goal -* Water framework directive**

Explanation: Directly deals with aquatic contamination, relevant due to the close interactions between water and sediment. Important since sediment might indicate long-term representation of an area while water changes rapidly. Sediment should receive more attention in the work related to the water framework directive.

***Goal -* Bathing water directive**

Explanation: The directive deals with the status of water at places used for recreational outdoor bathing. However, the focus seems overly limited to bacteria and contaminants might be overlooked.

#### Part 3. Does the interviewee agree to the notion that the additional goals suggested by other respondents to the survey are relevant to management of contaminated sediments? If so, what connection do the interviewee see?

In response to the survey, additional goals were suggested by other respondents. Are the suggested goals relevant in relations to contaminated sediments according the interviewee? If so, what is the connection according to the interviewee?

***Goal -* Zero hunger**

Response: Clear connection when contaminants affect food availability and opportunities for work.

***Goal -* No poverty**

Response: Relevant in case fishing is important as a means of income or food.

***Goal -* Quality education**

Response: No direct connection.

***Goal -* Gender equality**

Response: No direct connection.

***Goal -* A non-toxic environment**

Response: Less relevant than it appears. The introduction to the goal has a clear connection which is not reflected in the rest of the objectives texts.

***Goal -* National regulation for dumping waste at sea**

Response: We are not allowed to dump contaminated sediments at sea. Lacking knowledge of where dumping has occurred.

***Goal -* EU strategy for the Baltic Sea Region**

Explanation: A clean marine environment is part of the goal. Focus on economic aspects and partnership.

***Goal -* Baltic Sea Action Plan**

Response: Related to the Baltic Sea Action Plan but with a larger focus on research.

### Questionnaire and answers from interview 2. In Swedish.

#### Part 1. What is the connection between the directly relevant goals and management of contaminated sediments?

Notes from part 1 of the follow up interviews regarding the goals indicated as directly or partially relevant by the respondents. Each interviewee had earlier indicated whether the relation between each goal in the table to contaminated sediments was one of the following: Directly relevant, Partially relevant, Not relevant, or Unknown. The goals in part 1 are all either UN Sustainable development goals or Swedish National Environmental Objectives.

| ***Goal -* Good health and well-being** |
| --- |
| Directly relevant. Connections from edibles and the food web. Direct connection during e.g. swimming. |
| ***Goal -* Clean water and sanitation** |
| Directly relevant. Possible source for contaminants to drinking water. |
| ***Goal -* Life below water** |
| Directly relevant. An obvious and important goal. Healthy marine ecosystem as a resource, e.g. fishing. |
| ***Goal -* Life on land** |
| Directly relevant. Ecosystem quality. Earlier limited focus on water has broadened to include sediment, the benthic fauna and the food web. An important area is the zone between land and sea. Contaminants affect which organisms thrive in the sediment. Changes in the ecosystem such as boat traffic, changes in input of new sedimenting material from e.g. dredging or damming and erosion protection. The importance of the goal can be very large depending on the definition of sediment. |
| ***Goal -* Flourishing lakes and streams** |
| Directly relevant. Poor sediment quality has an obvious effect on the water and ecosystems. Should be more focus on identifying how we can determine when an area is unaffected rather than affected. |
| ***Goal -* A balanced marine environment, flourishing coastal areas and archipelagos** |
| Directly relevant. Similar to the objective of Flourishing lakes and streams but with a marine focus. |
| ***Goal -* A rich diversity of plant and animal life** |
| Directly relevant. Overlaps with Life on land. Contaminants can affect organisms and biodiversity. Clearly relevant for aquatic environments. |
| ***Goal -* Decent work and economic growth** |
| Partially relevant. Risks when in contact with contaminants during work in relation to sediment. Effects on the possibility to use ecosystems for economic development, e.g. tourism, recreation, fishing. |
| ***Goal -* Good-quality ground water** |
| Partially relevant. Mainly in situations when groundwater interact with surface water through seepage. Also relevant in flood events when sediment contaminants spread to surface water covering land and seep into the ground water. |
| ***Goal -* Reduced climate impact** |
| Directly relevant. Relevant during management of contaminated sediments. Resources used during management. Does not include the impacts of climate change on management methods and practice. Always necessary to consider climate, changing or not, we already have weather affecting sediments. |
| ***Goal -* Sustainable cities and communities** |
| Partially relevant. The quality of life connects to clean water, healthy ecosystems. It also connects to how we build and develop our communities in relation to effects on and from contaminated sediment. Society is a source for sediment contamination. |
| ***Goal -* Thriving wetlands** |
| Partially relevant. Contaminants might reach wetlands during flooding events from sediments and contaminants and material can move from wetlands to sediments. Not a clear or general connection. |
| ***Goal -* Good built environment** |
| Partially relevant. Similar to the goal Sustainable cities and communities. Two dimensions. The community should not negatively affect the sediment and vice versa. |
| ***Goal -* Industry, innovation and infrastructure** |
| Partially relevant. Industry as a source of contaminants. We need this goal to stop continuous contamination. |
| ***Goal -* A varied agricultural landscape** |
| Partially relevant. Two dimensions: aquaculture can be release contaminants to, and receive contaminants from, sediments. Agriculture on land is a potential source of contaminants and potentially at risk from sediments during flooding events. |
| ***Goal -* Climate action** |
| Partially relevant. Similar to the objective “Reduced climate impact” in terms of how it relates to the use of resources. |

#### Part 2. What is the connection between the partially relevant goals and management of contaminated sediments?

The respondent is asked to explain the connection between contaminated sediment and additional goals the respondent suggested were relevant when answering the survey.

***Goal -* No Poverty**

Explanation: Less relevant in a Swedish context. Aquatic resourrses such as fish are an important source of income globally. Sediment can be used in development and construction but connected to risks if it is contaminated. Fishing would perhaps be a larger field of work if the Baltic had been less polluted.

***Goal -* Equal education**

Explanation: Can be connected to an understanding of the consequences of polluting and following choices not to pollute.

***Goal -* Zero hunger**

Explanation: Aquatic environments as producers of food stuff. Not very relevant in a Swedish context. Local problems in other countries. Important to ensure that the environment will be able to provide food stuff in the future when the needs and demands might differ from now. Important already as we are missing out on an important source of nutrients.

***Goal -* Gender equality**

Explanation: Unequal that people have differences in health depending on where they live in relation to contaminants. Unequal that wealthier communities can afford to manage contaminants where poorer communities can not.

#### Part 3. Does the interviewee agree to the notion that the additional goals suggested by other respondents to the survey are relevant to management of contaminated sediments? If so, what connection do the interviewee see?

In response to the survey, additional goals were suggested by other respondents. Are the suggested goals relevant in relations to contaminated sediments according the interviewee? If so, what is the connection according to the interviewee?

***Goal -* Water framework directive**

Answer: Obvious goal. To reach a good status as dictated in the directive we need to consider the sediments.

***Goal -* EU strategy for the Baltic Sea Region**

Answer: No clear connection.

***Goal -* Baltic Sea Action Plan**

Answer: No clear connection.

***Goal -* A non-toxic environment**

Answer. Obvious goal. The goal is not fullfilled if we have contaminated sediments.

***Goal -* Marine environment directive**

Answer: No clear connection.

### Questionnaire and answers from interview 3. In Swedish.

#### Part 1. What is the connection between the directly relevant goals and management of contaminated sediments?

Notes from part 1 of the follow up interviews regarding the goals indicated as directly or partially relevant by the respondents. Each interviewee had earlier indicated whether the relation between each goal in the table to contaminated sediments was one of the following: Directly relevant, Partially relevant, Not relevant, or Unknown. The goals in part 1 are all either UN Sustainable development goals or Swedish National Environmental Objectives.

| **Good health and well-being** |
| --- |
| Directly relevant. Contact with hazardous substances through the food web. |
| **Clean water and sanitation** |
| Directly relevant. Connection between sediments and drinking water. Sediment management risks spreading contaminants to drinking water. Climate change and land upheaval can increase the risk of contaminants spreading. |
| **Life below water** |
| Directly relevant. There is a direct connection between the sea and sediment. Broad definition of resources including recreation and foodstuff. Difficult to use those resources if there is a risk from the sediments. E.g. dredging for development and maintenance. Animals as a resource, foodstuff, makes the connection especially problematic. |
| **Life on land** |
| Directly relevant. Direct connection between the goal and contaminated sediments. Contaminants affecting organisms might lead to loss of species and ecosystem functions. The goal deals with ecosystem in place. |
| **Flourishing lakes and streams** |
| Directly relevant. Obvious connection as contaminated sediments might contaminate surrounding waters. Risk for transportation if sediments will release contaminants when dredged. Drinking water from lakes at risk. |
| **A balanced marine environment, flourishing coastal areas and archipelagos** |
| Directly relevant. Problematic with contaminated sediments and boat traffic. Difficult to boats access to contaminated areas, imperative to manage the risk from those areas. Historic and current activities put higher demands on the marine areas in relation to in land water bodies. Reduce access to the marine environment due to contamination might cause social and economic stress in surrounding land areas. Aspects such as recreation are important. |
| **A rich diversity of plant and animal life** |
| Partially relevant. Unclear to what extent the objective is relevant for aquatic environments. Contaminants in the sediment might still affect terrestrial organisms. |
| **Good-quality ground water** |
| Directly relevant. Increased costs if necessary to purify contaminated drinking water. Clear connection to humans through drinking water. |
| **Sustainable cities and communities** |
| Partially relevant. Important connection to boat traffic and recration which requires access to the marine environment without risk for contamination, e.g. during dredging to keep thoroughfares open. Costly to manage contaminated sediments, resources spent on sediment management unavailable for other use. Small recreational boat associations are allowed to continue their activities without paying for historical contamination. |
| **Good built environment** |
| Partially relevant. Similar to the goal Sustainable cities and communities. We want to be able to have recreational boat associations, necessitating clean sediment. Difficult for the associations to carry the costs of for remediation of contaminated sediments. |
| **Industry, innovation and infrastructure** |
| Partially relevant. Innovations and research are needed. Current practices and techniques are expensive and not efficient, causing unknown side effects, e.g. spread of contaminants during dredging. We need a better understanding of the effectiveness of current techniques are and lower costs. This goal dictates that we have to further innovation. |

#### Part 2. What is the connection between the partially relevant goals and management of contaminated sediments?

The respondent is asked to explain the connection between contaminated sediment and additional goals the respondent suggested were relevant when answering the survey.

**A non-toxic environment**

Explanation: Connected through the negative effects of environmental contaminants. Necessary to consider the contaminants in sediment and limit their spread. Antifouling paint from boats is and has spread extensively to sediments. Goals are opposing in this matter as the costs for management would limit the work towards other goals.

#### Part 3. Does the interviewee agree to the notion that the additional goals suggested by other respondents to the survey are relevant to management of contaminated sediments? If so, what connection do the interviewee see?

In response to the survey, additional goals were suggested by other respondents. Are the suggested goals relevant in relations to contaminated sediments according the interviewee? If so, what is the connection according to the interviewee?

**Zero hunger**

Answer: connected through ecosystem resources such as food and clean drinking water.

**No poverty**

Answer: Connected to sediment in other countries. Less so in Sweden as there are affordable alternatives.

**Equal education**

Answer: No clear connection

**Gender Equality**

Answer: No clear connection

**Water framework directive**

Answer: Directly relevant. The directive provides a basis for preventing and forbidding products which might hinder the aquatic environment from reaching a good status.

**Marine environment directive**

Answer: The same as for the Water framework directive but for other aquatic environments. The quality of aquatic environments may not be reduced. Management may not reduce environmental quality. Not even temporary reductions should be allowed.

**EU strategy for the Baltic Sea Region**

Answer: International platform for cooperation. Important for raising issues internationally and for governmental bodies to work together.

**Baltic Sea Action Plan**

Answer: No clear connection.
